# Unsaturated free fatty acid emulsion infusion into carotid artery enhances drug delivery to the rat brain

**DOI:** 10.1002/brb3.2994

**Published:** 2023-05-22

**Authors:** Yong‐Woo Kim, Seon Hee Choi, Ki Joo Choi, Hak Jin Kim, Jin Sook Kang, Dong Jin Hong

**Affiliations:** ^1^ Department of Radiology, College of Medicine Pusan National University, Research Institute for Convergence of Biomedical Science and Technology, Pusan National University Yangsan Hospital Busan South Korea; ^2^ Institute for Research and Industry Cooperation Pusan National University Busan South Korea; ^3^ MSD‐EM, Thermo Fisher Scientific Hillsboro Oregon USA; ^4^ Department of Radiology, College of Medicine Pusan National University, Pusan National University Hospital, Biomedical Institute of Pusan National University Hospital Pusan South Korea; ^5^ School of Mechanical engineering Pusan National University Busan South Korea; ^6^ Department of Information Convergence Engineering Pusan National University Busan South Korea

**Keywords:** antineoplastic drug, BBB, DESI–MS, electron microscopy, free fatty acid

## Abstract

**Aims:**

: To determine whether the blood–brain barrier (BBB) opens to enhance drug delivery during the acute stage of unsaturated fat embolism.

**Methods:**

: We infused oleic, linoleic, and linolenic acid emulsions through the right common carotid artery of rats, followed by trypan blue for gross and lanthanum for electron microscopic (EM) examination. Doxorubicin and temozolomide were also administered, and then the rats were euthanized at 30 min, 1 h, and 2 h. Trypan blue hue was analyzed to semiquantitatively measure BBB opening. Desorption electrospray ionization–mass spectrometry (DESI–MS) imaging was used to evaluate drug delivery.

**Results:**

Trypan blue staining observed in each group 30 min after emulsion infusion increased at 1 h and decreased after 2 h in the oleic acid group. The linoleic and linolenic acid groups showed weak staining over time. The hue and trypan blue analysis results were corroborative. EM showed tight junction opening, whereas DESI–MS imaging showed increased doxorubicin and temozolomide signal intensities in ipsilateral hemispheres of all three groups.

**Conclusion:**

We demonstrated that oleic, linoleic, and linolenic acid emulsions opened the BBB, promoting drug delivery to the brain. Hue analysis and DESI–MS imaging are appropriate for analysis of doxorubicin and temozolomide concentrations in brain tissue.

## Graphical abstract

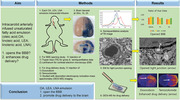

## 1 INTRODUCTION

The blood–brain barrier (BBB) is a structural and functional barrier between the blood and brain parenchyma. This barrier regulates brain homeostasis and transportation of endogenous and exogenous materials by controlling their selective and specific uptake, efflux, and metabolism in the brain. Consequently, various drug delivery and targeting strategies are currently being developed to overcome the limiting properties of the BBB (De Boer & Gaillard, 2007). Triolein, a major constituent of bone marrow fat, causes clinical fat embolism syndrome. Brain fat embolism syndrome causes neurological symptoms and signs in up to 100% of patients according to the amount of embolized fat (Bouaggad et al., 1995).

Clinical cerebral fat embolism differs from ischemic infarction in that it is self‐limiting. The silent symptoms of the disease make early diagnosis difficult (Sevitt, 1977). In experimental cerebral fat embolism studies using triolein, early vasogenic and cytotoxic edema were revealed using magnetic resonance imaging (MRI) (Kim et al., 2001). However, infusing triolein as an emulsion only transiently and reversibly opened the BBB opening infused into the carotid artery (Kim et al., 2004). The BBB opening time was evaluated densely in the hyperacute stage of fat embolism (<6 h after infusion) following triolein emulsion (Kim et al., 2004, 2006), free fatty acid (FFA) emulsions also opened the BBB, although the opening time was not evaluated thoroughly (Kim et al., 2005).

These fat emulsion techniques have focused on drug delivery enhancement. The widely used anticancer agents doxorubicin and temozolomide have targeted for brain delivery using the triolein emulsion technique (Kim et al., 2016; Seung et al., 2021). However, no studies have investigated the delivery of antineoplastic drugs using the FFA emulsion technique. Several experimental methods have been used to study BBB opening, such as MRI using contrast gadolinium and electron microscopy (EM) using contrast lanthanum nitrate, trypan blue, and Evans blue staining. These studies all investigated the leakage of contrast materials or stains through the BBB into the interstitium.

Desorption electrospray ionization–mass spectrometry (DESI–MS) imaging of biological tissues is an efficient and highly sensitive technique for imaging lipids and metabolites in biological tissues (Eberlin et al., 2011). DESI is clinically applicable because it enables the analysis of biomolecules in the *x*‐ and *y*‐axis directions by spraying charged droplets and provides chemical information that can be displayed as two‐dimensional (2D) images (Agar et al., 2011). Temozolomide has been shown to be efficiently delivered to the brain with the triolein emulsion technique using DESI–MS imaging (Seung et al., 2021). However, DESI–MS imaging has not been used to enhance the delivery of antitumor agents using the FFA emulsion technique. The purpose of the present study was to evaluate whether the BBB opens during the hyperacute stage of fat embolism following the infusion of emulsions of three unsaturated FFAs (oleic, linoleic, and linolenic acids) into the carotid artery of rats using trypan blue staining and transmission EM (TEM), and drug delivery of doxorubicin and temozolomide was evaluated using DESI–MS imaging.

## 2 METHODS

### 2.1 Animal model preparation

**FIGURE 1:**
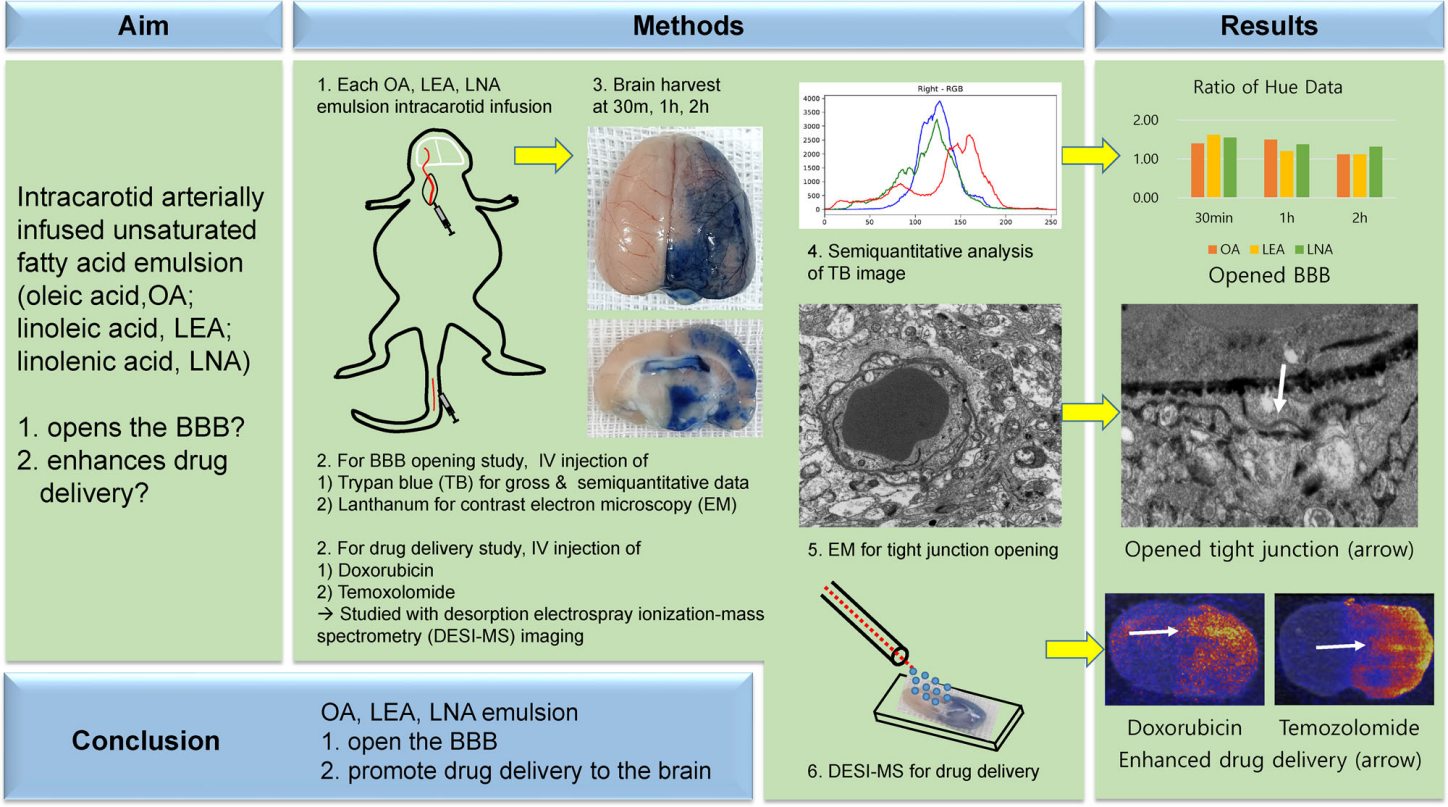
Graphical Abstract. We infused each free fatty acid (FFA; oleic acid, OA; LEA, linoleic acid; LNA, linolenic acid) intracarotid arterially, followed by trypan blue for gross and lanthanum for EM (electron microscopy), and anticancer agents (doxorubicin and TMZ, temozolomide) intravenously. Desorption electrospray ionization–mass spectrometry (DESI–MS) imaging was used to evaluate drug delivery. The brain tissues were harvested at 30 min, 1 h, and 2 h. Trypan blue Joint Photographic Experts Group (JPG) images and their hue were measured blood–brain barrier (BBB) opening qualitatively and semiquantitatively, respectively. Trypan blue staining observed in each group. The hue and trypan blue analysis results were corroborative. EM showed tight junction opening, DESI–MS imaging showed increased drug signal intensity in ipsilateral hemispheres of all three groups. We demonstrated that oleic, linoleic, and linolenic acid emulsions opened the BBB and promoted drug delivery to the brain.

The Institutional Animal Review Board of our Biomedical Research Institute approved all the experimental protocols (Approval No: PNUH‐2016–086). Experiments were performed using 14‐week‐old adult male Sprague–Dawley rats (KOATECH, Gyeonggi‐do, Korea) weighing approximately 300 g after 1 week of acclimation. All animals were kept in a semi‐specific pathogen‐free environment maintained at 18–22°C under a 12‐h light/dark cycle and were allowed free access to water and food ad libitum. Animals were anesthetized using an intramuscular injection of ketamine hydrochloride (HCl, 2.5 mg/kg; Huons, Chungcheongbuk‐do, Korea) and xylazine (0.125 mg/kg; Bayer Korea, Seoul, Korea) and allowed to breathe ambient air spontaneously during the procedure. The experimental protocol used in this study is described schematically in Figure 1 (graphical abstract).

### 2.2 Infusion of FFA emulsions

The right common, internal, and external carotid arteries were isolated, and the occipital artery was ligated using surgical microscopy. A 20 gauge intravenous catheter (Angiocath, Becton Dickinson, Sandy, UT, USA) was inserted into the common carotid artery. Then, 1 mL syringes containing 100 μL oleic, linoleic, or linolenic acids (Sigma‐Aldrich, MO, USA), and 10 mL syringes containing 10 mL normal saline were connected to each three‐way stopcock. Each FFA emulsion was prepared by mixing the FFA with normal saline using a vigorous to‐and‐fro movement of the syringes for 3 min. Each FFA emulsion (2 mL of a 10% emulsion) was then infused manually into the rat brain via the inserted catheter over 1 min.

### 2.3 Gross and histologic evaluation

For gross evaluation of the BBB opening, trypan blue (2 mL, Sigma‐Aldrich, MO, USA) was injected intra‐arterially 30 min, 1 h, or 2 h after FFA or triolein emulsion infusions. For the EM evaluation, 2 mL 5% lanthanum nitrate (EMS, PA, USA) was used as an EM contrast agent and was injected intravenously through the tail vein after completing each time course (30 min, 1 h, and 2 h).

The animals were immediately euthanized using carbon dioxide (CO_2_) gas, and the brain tissues were excised, whereas the whole brain was photographed for complete visualization. Thin, 1 mm thick sections were prepared using a rat brain cutting matrix (Kent Scientific Corp, Torrington, CT, USA), and each section was photographed again. Images were saved as Joint Photographic Experts Group (JPG) files. For semiquantitative analysis of the BBB opening visualized as a blue staining with trypan blue, the JPG files were analyzed using a Python program language code. The color of the brain was compared 30 min, 1 h, and 2 h after treatment. The red, green, and blue (RGB) values of the image on the ipsilateral (treated) and contralateral hemispheres were extracted for the specific time, and a histogram was drawn to confirm the direction of the color change. The hue data were obtained from the RGB data to calculate the mean and standard deviation of each image at different times.

For EM evaluation, brain tissue samples from the trypan blue‐stained area were immediately cut into 5 × 5 mm pieces, placed in a solution of 2.5% glutaraldehyde and 2% paraformaldehyde in 0.1 M phosphate buffer at pH 7.4, and left to soak for 2 h at 4°C. The specimens were then rinsed with 0.1 M phosphate buffer three times at 10 min intervals. Specimens were then postfixed in 1% osmium tetroxide (OsO_4_; EMS, PA, USA) 0.1 M phosphate buffer in the dark rinsed in 0.1 M phosphate buffer for 10 min, dehydrated in a graded ethanol series, and embedded in Epon epoxy resin (EMS, PA, USA). Ultrathin sections were cut using a knife (UC7, Leica, Wetzlar, Germany) and examined using EM (Tecnai G^2^ Spirit Twin, FEI, USA). EM was mainly used to examine lanthanum nitrate leakage into the interstitium through the BBB.

### 2.4 Drug delivery and qualitative analysis using DESI–MS imaging

For qualitative analysis of drug delivery enhancement, doxorubicin (2.4 mg/kg) or temozolomide (18 mg/kg) was injected after infusing oleic, linoleic, or linolenic acid emulsions into the carotid artery. Then, 1 h later, the rats were euthanized with CO_2_ gas, and the brain tissues were harvested and coronally sectioned for DESI–MS imaging.

DESI–MS imaging was performed using a Waters XEVO G2‐XS quadrupole time‐of‐flight (Q‐ToF) mass spectrometer (Waters, Milford, MA, USA). The DESI ion source was mounted on a mass spectrometer and controlled using Omni Spray software (Prosolia, Indianapolis, IN, USA). The DESI source was initially set up using a rock spray solvent of 0.1% formic acid in acetonitrile:water (95:5, v/v), and 0.2 ng/μL leucine enkephalin (*m*/*z* 556.2771 in the ESI+ mode) was added as the internal standard and lock mass compound.

The flow rate was 2 μL/min in the positive ion mode. All MS parameters were recorded under the following conditions: capillary voltage, 5 keV; sample cone voltage, 40 V; source and desolvation temperatures, 150 and 250°C, respectively; desolvation and cone gas flow rates, 600 and 50 L/h, respectively; gas pressure, 4.5 bar; spray voltage, 4.5 kV; spray, sprayer incidence, and collection angles, 60°, 75°, and 10°, respectively; sprayer‐to‐inlet and sprayer‐to‐sample distances, 3 and 1 mm, respectively; source temperature, 150°C; and source offset, 80 V.

Prior to image acquisition, the detected ion intensity of the red Sharpie marker pen (rhodamine) [M + H]^+^ at *m*/*z* 443.23 for the positive ion mode was verified. The emitter capillary protrusion was initially optimized by observing the removal of the material from an ink patch on a glass slide. Mass spectra were acquired at an *m*/*z* range of 50–700 for all MS analyses, and 1000 of the most intense peaks were observed. All *m*/*z* values were extracted in a mass window of 0.02 Da, and the total scan time was determined based on the pixel size and scan speed.

The square pixel size was 100 μm for the MS imaging, the scan speed was 100–110 μm/s, and the time required for imaging brain samples was 230–240 min. All imaging data were acquired and analyzed directly using high‐definition imaging (HDI) version 1.4 in combination with MassLynx version 4.1 (Waters, Milford, MA, USA). The HDI software enabled the acquisition and processing of data from DESI–MS imaging experiments. TMZ was identified at *m*/*z* 217 because of the sodium adduct ion [M + Na]^+^ 194 → 217, whereas doxorubicin was detected at *m*/*z* 544 [M + H]^+^ 543 → 544 in the positive mode.

## 3 RESULTS

**FIGURE 2:**
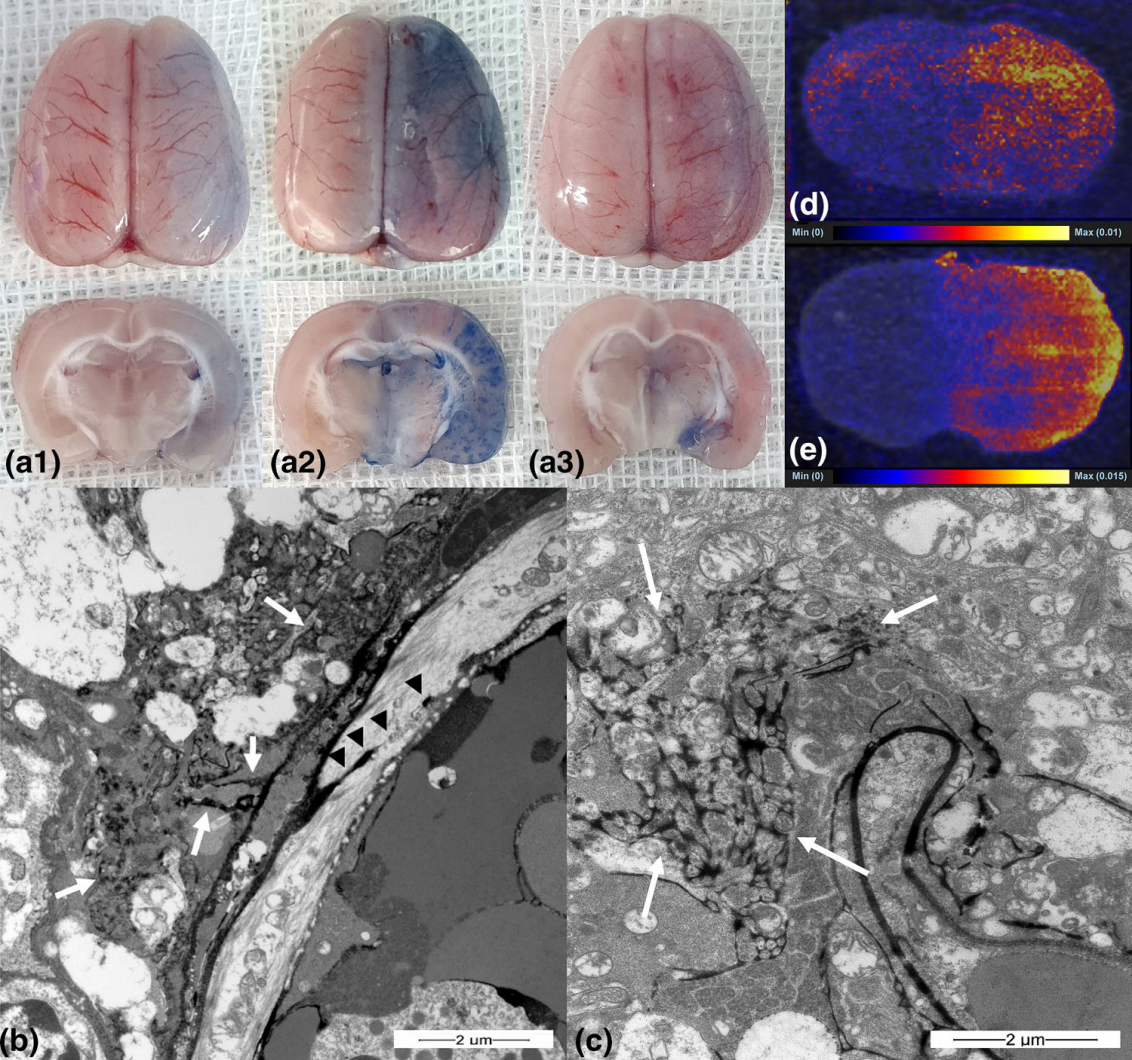
Gross finding in trypan blue‐stained whole brain (a1, b1, and c1) and brain tissue sections (a2, b2, and c2) in oleic acid group (A). Blue staining in ipsilateral hemisphere due to blood–brain barrier opening is seen from (a1 and a2) 30 min after infusion of oleic acid emulsion into carotid artery, which increased at 1 h (b1 and b2) and decreased at 2 h (c1 and c2). Electron microscopy examination (×12,000) 1 h after infusion of oleic acid emulsion into the carotid artery shows tight junction opening (B, arrow heads) and interstitial spillage (arrows) of lanthanum (B, C, arrows). Desorption electrospray ionization–mass spectrometry (DESI–MS) image of doxorubicin 2.4 mg/kg injection after oleic acid emulsion infusion into carotid artery (D). Ipsilateral hemisphere shows higher signal intensity than that of contralateral hemisphere. DESI–MS image following temozolomide 18 mg/kg injection after oleic acid emulsion infusion into carotid artery (E). Ipsilateral hemisphere shows higher signal intensity than that of contralateral hemisphere.

**FIGURE 3:**
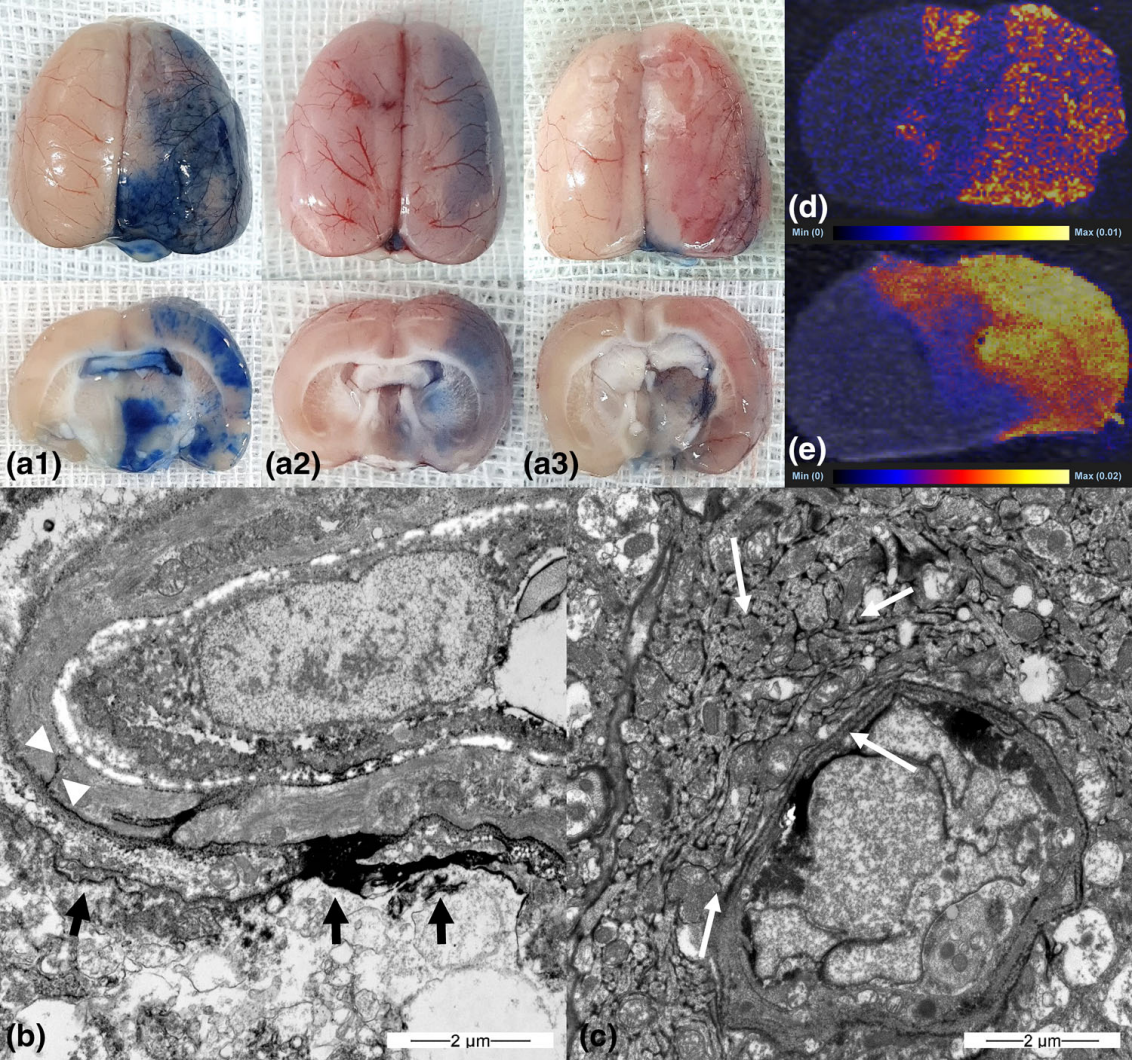
Gross finding of trypan blue‐stained whole brain (a1, b1, and c1) and brain tissue sections (a2, b2, and c2) in linoleic acid group. Blue staining in ipsilateral hemisphere shows that blood–brain barrier opening is seen from 30 min (a1 and a2) after infusion of linoleic acid emulsion into carotid artery, which later decreased at 1 h (b1 and b2) and 2 h (c1 and c2). Electron microscopy examination (B, ×15,000; C, ×12,000) 30 min after infusion of linoleic acid emulsion into the carotid artery shows tight junction opening (B, arrow heads) and interstitial spillage of lanthanum (B, C, arrows). Desorption electrospray ionization–mass spectrometry (DESI–MS) image following injection of doxorubicin 2.4 mg/kg after linoleic acid emulsion infusion into carotid artery (D). Signal intensity is higher in ipsilateral hemisphere than it is in contralateral hemisphere. DESI–MS image following injection of temozolomide 18 mg/kg after linoleic acid emulsion infusion into carotid artery (E). Signal intensity is higher in the ipsilateral hemisphere than in the contralateral hemisphere.

**FIGURE 4:**
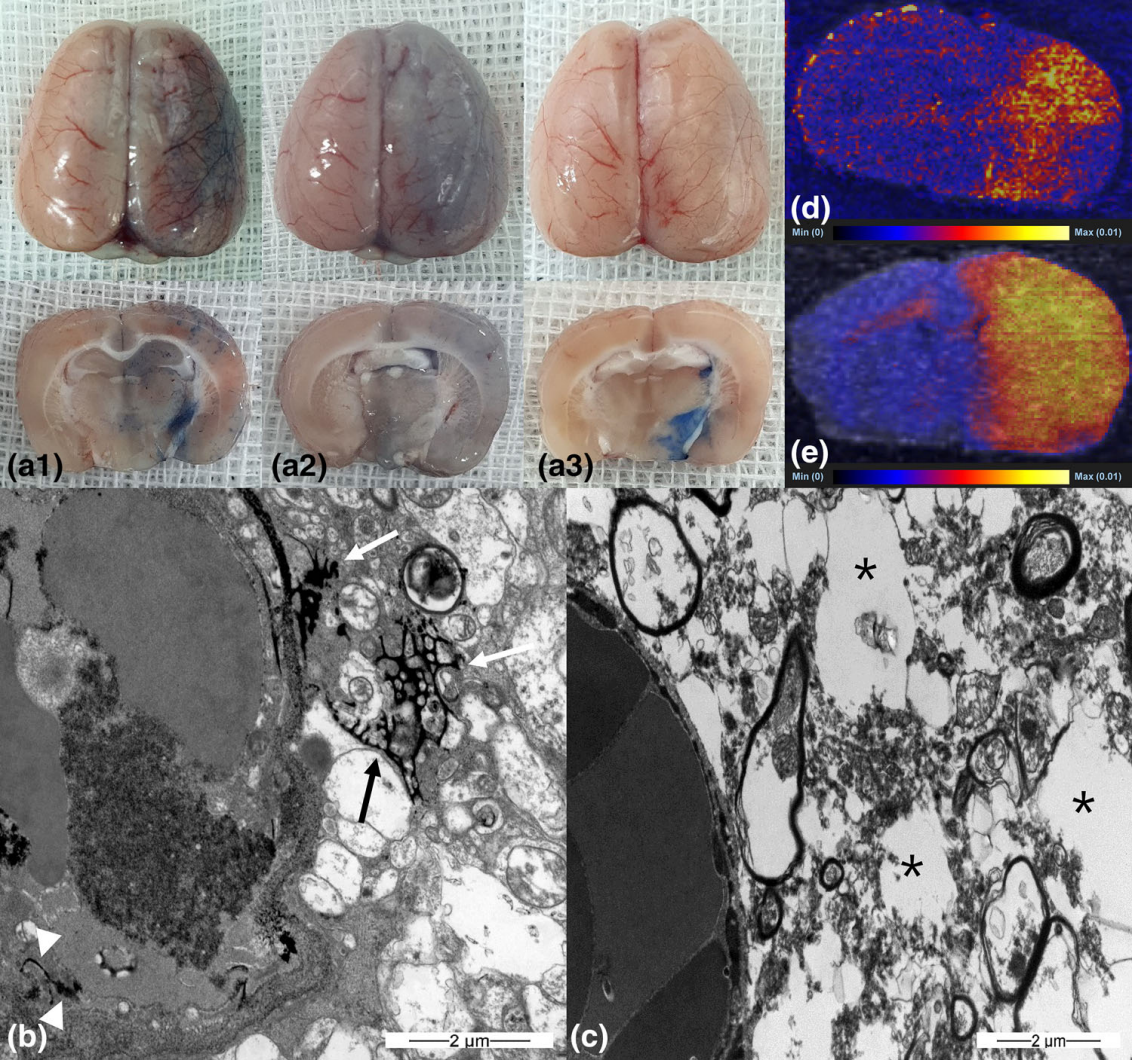
(A) Gross finding of trypan blue‐stained whole brain (a1, b1, and c1) and brain tissue sections (a2, b2, and c2) in linolenic acid group. Blue staining in ipsilateral hemisphere indicates blood–brain barrier opening from 30 min (a1 and a2) after infusion of linolenic acid emulsion into the carotid artery, which decreased at 1 h (b1 and b2) and 2 h (c1 and c2). Electron microscopy examination (B, ×15,000; C, ×12,000) 1 h after linolenic acid emulsion infusion into the carotid artery shows tight junction opening (B, arrow heads) and interstitial space filled with lanthanum (B, arrows). Widening of the perivascular interstitial space (C, asterisks) is also shown. Desorption electrospray ionization–mass spectrometry (DESI–MS) image following injection of doxorubicin 2.4 mg/kg after linolenic acid emulsion infusion into carotid artery (D). Ipsilateral hemispheric signal intensity is stronger than that of contralateral hemisphere. DESI–MS image following injection of temozolomide 18 mg/kg after linolenic acid emulsion infusion into carotid artery (E). Ipsilateral hemispheric signal intensity is stronger than that of contralateral hemisphere.

Blue staining by trypan blue indicated that BBB opening occurred in all three groups 30 min after emulsion infusion. The blue staining was stronger at 1 h (*n* = 5) and weaker at 2 h (*n* = 5) in the oleic acid group (Figure 2A). The linoleic and linolenic acid groups showed weaker staining over time (1 and 2 h, *n* = 5 in each group; Figures 3A and 4A). The EM evaluation showed that the tight junctions were filled with contrast medium (lanthanum) at a width of 10–47 nm (Figures 2, 3, and 4B), and lanthanum leakage into the interstitium was observed in all three groups 30 min and 1 h after emulsion infusion (Figures 2C and 3C). However, leakage was seldom observed 2 h after emulsion infusion. Widening of the perivascular interstitial space was more frequently observed after 30 min, 1 h, and 2 h in the EM analysis of the linolenic acid group (Figure 4C). DESI–MS imaging showed that the signal intensities of doxorubicin (Figures 2, 3, and 4D) and temozolomide (Figures 2, 3, and 4E) were remarkably higher in the ipsilateral hemisphere than in the contralateral hemisphere in all the three FFA groups.

### 3.1 Hue data for semiquantitative analysis of the BBB opening

**FIGURE 5:**
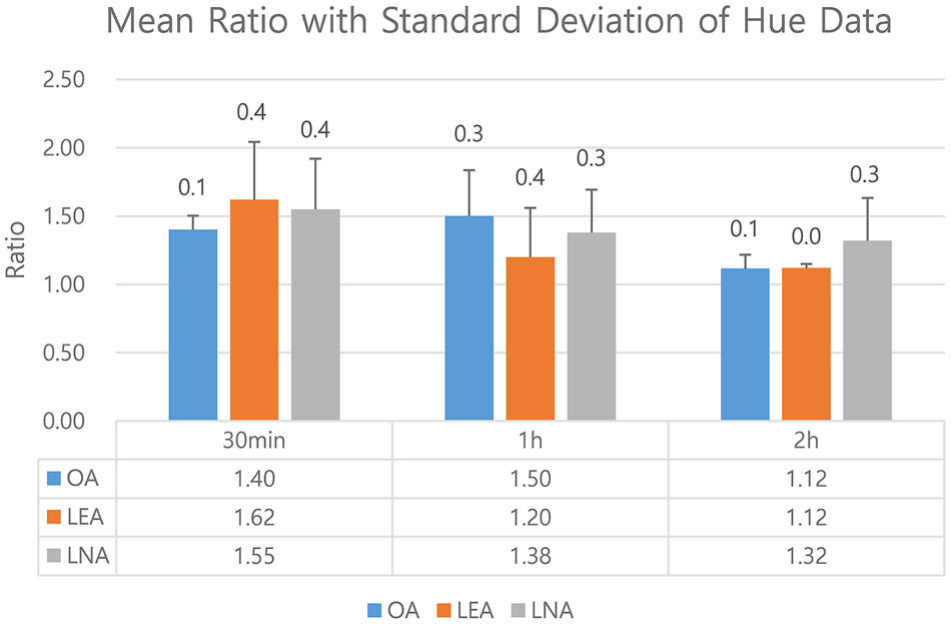
Graph of ratio of hue data in ipsilateral hemisphere contrast to that in contralateral hemisphere. Higher value >1.0 indicates stronger trypan blue staining due to opening of blood–brain barrier in ipsilateral hemisphere. Linoleic and linolenic acid emulsion groups show highest staining at 30 min, which decreased after that. Oleic acid emulsion group shows highest staining at 1 h.

The average values of the corrected hue data of the oleic, linoleic, and linolenic acid‐, and triolein‐treated groups at specified times and the ratio of the average values are shown in Figure 5. The corrected hue data were as follows: 99‐H (0 ≤ H < 100), 459‐H (100 ≤ H <360), and H: real hue data. In the oleic acid group, the ratio of the average image analysis data was 1.40 at 30 min, which increased at 1 h and then decreased at 2 h. In the linoleic and linolenic acid groups, the ratio of the average image analysis data was highest at 30 min and then it decreased (Figure 5).

## 4 DISCUSSION

The major finding of the present study is that all three unsaturated FFA emulsions opened the BBB immediately after intra‐arterial infusion into the carotid artery as indicated by the EM, trypan blue staining, and hue measurement analyses. This finding was also proven by the increased intraparenchymal delivery of the anticancer agents (doxorubicin and temozolomide) injected after the emulsion infusion in all three unsaturated FFA groups, which was evaluated using DESI–MS imaging. A previous EM study showed that oleic and linoleic acids disrupted the BBB when they were infused into the carotid artery as emulsions (Kim et al., 2005). However, morphologically confirmed opening of tight junctions by the three unsaturated FFA emulsions revealed in the present study has not been previously studied. This paracellular opening appears to be a key feature of the morphological mechanism by which these three unsaturated FFAs affect the BBB, which is the molecular mechanism we investigated. In the present study, the linolenic acid emulsion group showed more frequent widening of the perivascular space, which suggested more damage than that in the oleic or linoleic acid emulsion groups. BBB opening and brain tissue damage might depend on the amount and kind of fat. The higher the amount of infused fat, the larger the BBB opening or tissue damage. Further studies are needed to compare the BBB opening effects of different types of fat.

Doxorubicin cannot cross the BBB (Stan et al., 1999; Von Holst et al., 1990). Temozolomide can penetrate the BBB. The penetration of orally or intravenously administered temozolomide into the brain has been shown to vary (20%–39%) based on the brain/plasma AUC ratio (Ostermann et al., 2004). In the present study, examination of the DESI images showed no visible delivery of doxorubicin or temozolomide to the contralateral hemisphere.

In the present study, hue analysis was performed after changing the JPG files to RGB data using Python code, which is a popular computer programming language. This was because semiquantitative numeric analysis could be achieved from qualitative data of trypan blue staining, and then, a better understanding of the time‐series images of trypan blue‐stained gross figures. All three unsaturated fatty acid groups showed strong trypan blue staining and hue data measurement in the ipsilateral hemisphere 30 min after emulsion infusion and became weaker staining thereafter. Thus, BBB opening seems to start just after the infusion of unsaturated fatty acid emulsion and to decrease over time. However, peak trypan blue staining was noted at 1 h in the oleic acid group, probably due to increased BBB opening.

DESI–MS imaging is an emerging and useful technique for imaging lipids and metabolites in biological tissues (Eberlin et al., 2011). This imaging technique analyzes biomolecules in the *x*‐ and *y*‐axis directions by spraying charged droplets and then providing chemical information with 2D images (Agar et al., 2011). The advantages of this technique are its time efficiency, high discriminatory power, sensitivity, specificity, ability to analyze specimens in a wide mass spectrum, and efficient surface analysis (Cooks et al., 2006; Lostun et al., 2015; Takáts et al., 2004). In this study, the delivery of doxorubicin and temozolomide were observed to be promoted in the ipsilateral hemisphere through the opened BBB using DESI–MS imaging as an objective and geographical presentation. Furthermore, this opening of the BBB was achieved by infusing the three FFA emulsions into the carotid artery.

Unsaturated FFAs are fatty acids containing one or more double/triple carbon–carbon bonds in the carbon chain. Vegetables, legumes, nuts, seeds, and oils derived from these foods mostly consist of unsaturated fat. Oleic acid is the most important constituent of human fat (Peltier, 1956). In contrast, animal fats and coconut oil tend to be solid at room temperature because they are mostly saturated fats, which have no double bonds between carbon atoms. Dietary saturated fatty acids (SFA) can be grouped as short‐ and medium‐chain SFA (C2:0 to <C16:0) and long‐chain SFA (>16:0), depending on the carbon chain length. Milk and dairy products are the major sources of short‐ and medium‐chain fatty acids, whereas meat, processed meat products, and vegetable shortenings/partially hydrogenated vegetable oil are sources of long‐chain SFA. Neutral fats, also known as true fats, are simple lipids that are produced by the dehydration of one or more fatty acids with an alcohol, such as glycerol. Human fat derived from long bones and subcutaneous tissue is almost entirely composed of neutral fat (triolein). Irrespective of the location from which they are obtained, human fat cells are composed almost entirely of pure triglycerides.

Triolein is a major constituent of embolized fat in patients with fat embolism syndrome. Experimental cerebral fat embolism induced by a bolus injection of triolein induced cytotoxic edema due to vascular occlusion and vasogenic edema due to endothelial damage (Kim et al., 2001, 2002). However, cerebral fat embolism induced by triolein emulsion causes vasogenic edema only, which is reversible and transient (Kim et al., 2004). Thus, the type of embolized lesion may depend on the size of the fat globules. Bolus‐injected fat can obstruct blood vessels, resulting in brain infarction, whereas emulsified fat globules are much smaller and, therefore, do not occlude vessels (Kim et al., 2004). Reversible and transient BBB opening using the triolein emulsion infusion technique may be useful for enhancing drug delivery to the brain. In fact, anticancer agents were delivered using the triolein emulsion technique in an experimental brain model (Kim et al., 2016; Lee et al., 2020; Seung et al., 2021). Recently, Seung et al. (2021) demonstrated increased delivery of temozolomide with the aid of triolein emulsion using DESI–MS imaging in the rat brain.

The molecular mechanism of BBB opening by fat emulsion remains unknown. The possible mechanisms underlying fat embolism syndrome include mechanical and biochemical theories. The mechanical theory suggests that triglyceride particles from injured adipose tissue enter the circulation and obstruct the pulmonary vessels. The biochemical hypothesis implicates FFAs, suggesting that local hydrolysis of triglyceride emboli by pneumocyte lipase, together with excessive mobilization of FFAs from the peripheral adipose tissue by stress hormones, results in toxic concentrations of these acids in the lungs (Nakata et al., 1999). However, the morphological mechanism by which triolein emulsion (not bolus) opens the BBB mainly involves tight junction (Sol et al., 2017).

## 5 CONCLUSION

The results of the present study demonstrate that emulsions of oleic, linoleic, and linolenic acids promoted drug delivery to the brain and, thus, provide a new research tool for establishing strategies to enhance the therapeutic effects of anticancer agents. Additionally, hue analysis and DESI–MS imaging were demonstrated to be appropriate for the semiquantitative and qualitative analysis of doxorubicin and temozolomide concentrations in brain tissue and are worth further investigation for widespread application.

## AUTHOR CONTRIBUTIONS

Hak Jin Kim developed and designed the study concept; Yong‐Woo Kim and Hak Jin Kim wrote the initial draft; Seon Hee Choi, Ki Joo Choi, Jin Sook Kang, and Dong Jin Hong collected the data.

## CONFLICT OF INTEREST STATEMENT

The authors have no conflict of interests.

### PEER REVIEW

The peer review history for this article is available at https://publons.com/publon/10.1002/brb3.2994.

## Data Availability

Data are available upon request from the corresponding author.
